# Supplementary material for: The RAD51 recombinase protects mitotic chromatin in human cells
Source: Nat Commun. 2021 Sep 10;12:5380. doi: 10.1038/s41467-021-25643-y (PMC8433380; doi:10.1038/s41467-021-25643-y)
Supplement: Supplementary file 1 — Supplementary Information [file 41467_2021_25643_MOESM1_ESM.pdf]

Supplementary Information

The RAD51 recombinase protects mitotic chromatin in human cells

Isabel Wassing[1], Emily Graham[1], Xanita Saayman[1#], Lucia Rampazzo[1#], Christine Ralf[1],

Andrew Bassett[2], Fumiko Esashi[1*]

1. Sir William Dunn School of Pathology, University of Oxford, Oxford, UK

2. Wellcome Sanger Institute, Cambridge, UK

[#] These authors contributed equally

* Correspondence to: fumiko.esashi@path.ox.ac.uk

Index

Supplementary Figures

Supplementary Fig. 1: Evaluation of DNA synthesis upon mild replicative stress in mitosis.
Supplementary Fig. 2: Evaluation of RAD51 inhibitor B02 and conditional expression of RAD51 variants.
Supplementary Fig. 3: The impact of RAD51 and RAD52 depletion on MiDAS.
Supplementary Fig. 4: Evaluation of endogenous *RAD51* knock-in mutation at S14.
Supplementary Fig. 5: Synchronization of *RAD51* S14 knock-in mutant cell lines.
Supplementary Fig. 6: Evaluating mitotic progression.
Supplementary Fig. 7: Gating strategy.

Supplementary Tables

Supplementary Table 1: ssDNA template used to edit *RAD51* gene by CRISPR/Cas9
Supplementary Table 2: Primers used to screen *RAD51* knock-in mutation at S14

Supplementary Methods

Supplementary Reference

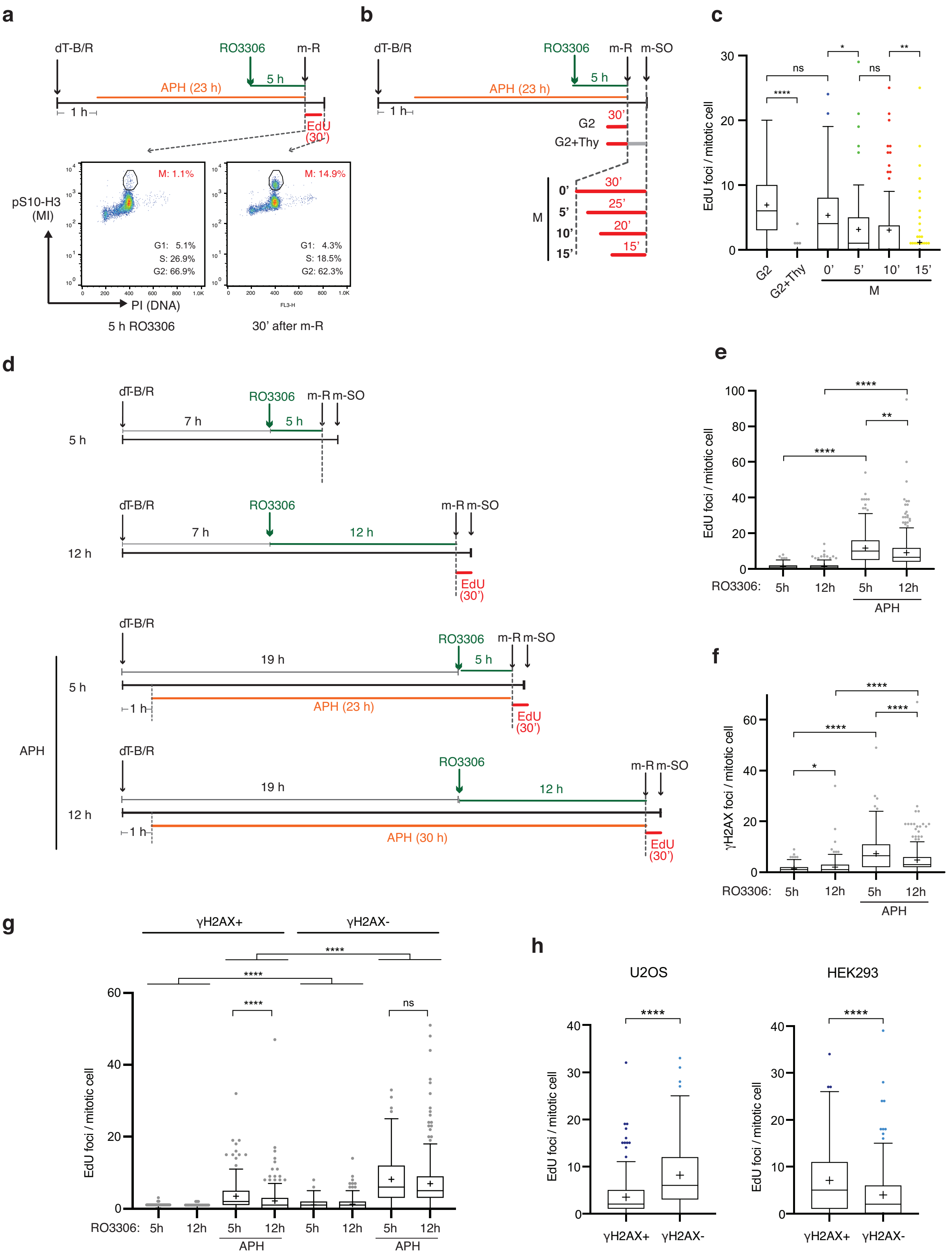

Supplementary Fig. 1: Evaluation of DNA synthesis upon mild replicative stress in mitosis.
a. Schematic diagram of experimental procedure for U2OS cell synchronisation. FACS profiles showing mitotic index (pS10-H3) and DNA content (PI) of cells exposed to RO3306 for 5 hours (left) and at 30 min after mitotic release (m-R) from RO3306 block (right) are also shown. The G1, S and G2 populations were estimated by the Watson Pragmatic algorithm (FlowJo), based on the PI staining of interphase cells. **b.** Schematic diagram of experimental procedure, as in (a), combined with 30 min EdU-labelling prior to m-R (G2), 30 min EdU-

labelling prior to m-R followed by a 30-min 100 μM thymidine chase upon m-R (G2+Thy); 30 min EdU-labelling upon m-R (M, 0'); 25 min EdU-labelling at 5 min upon m-R (M, 5'); 20 min EdU-labelling at 10 min upon m-R (M, 10'); and 15 min EdU-labelling at 15 min upon m-R (M, 15'). Cells were collected 30 min after m-R by mitotic shake-off (m-SO) and analysed for EdU incorporation. **c.** Quantification of the number of EdU foci per mitotic cell following EdU-labelling protocol as in (b). 100 cells were quantified per condition. Data distribution is represented by Tukey box-and-whisker plots. Bounds of box are 25–75th percentile, center shows the median and '+' marks the mean. Whiskers indicate ±1.5xIQR, data outside this range are drawn as individual dots. *p*-values were calculated by a Kruskal-Wallis test followed by Dunn's multiple comparison test. Asterisks indicate *p value ≤ 0.05; **p value ≤ 0.01; ****p value ≤ 0.0001. **d.** Schematic illustration of cell synchronisation by dT-B/R in the absence or presence of 0.4 μM APH and subsequent incubation in 9 μM RO3306 for 5 h or 12 h. Cells were labelled with EdU for 30 min upon m-R, collected by m-SO and stained for EdU and γ-H2AX. **e-f.** Quantifications of the number of EdU foci (e) and γ-H2AX foci (f) per mitotic cell. Data was obtained from 3 independent experiments (100 cells analysed per repeat); n=300 per condition. Data distribution is represented by Tukey box-and-whisker plots. Bounds of box are 25–75th percentile, center shows the median and '+' marks the mean. Whiskers indicate ±1.5xIQR, data outside this range are drawn as individual dots. *p*-values were calculated by a Kruskal-Wallis test followed by Dunn's multiple comparison test. Asterisks indicate *p value ≤ 0.05; **p value ≤ 0.01; ***p value ≤ 0.001; ****p value ≤ 0.0001. **g.** Quantification of the number of EdU foci that colocalise with γ-H2AX (γ-H2AX$^+$) and the number of EdU foci that do not colocalise with γ-H2AX (γ-H2AX$^-$) per mitotic cell. Data was obtained from 3 independent experiments (100 cells analysed per repeat); n=300 per condition. *p*-values were calculated by a Kruskal-Wallis test followed by Dunn's multiple comparison test. Asterisks indicate ****p value ≤ 0.0001. **h.** Mitotic EdU incorporation and γ-H2AX IF signal was analysed in U2OS and HEK293 cell lines following cell synchronisation in the presence of 0.4 μM APH and 5 hour RO3306-incubation. Quantifications of the number of EdU foci that colocalise with γ-H2AX (γ-H2AX$^+$) and the number of EdU foci that do not colocalise with γ-H2AX (γ-H2AX$^-$) per mitotic cell are shown. Data was obtained from 3 independent experiments (100 cells analysed per repeat); n=300 per condition. *p*-values were calculated by a Kruskal-Wallis test followed by Dunn's multiple comparison test. Asterisks indicate ****p value ≤ 0.0001. Source data are provided as a Source Data file.

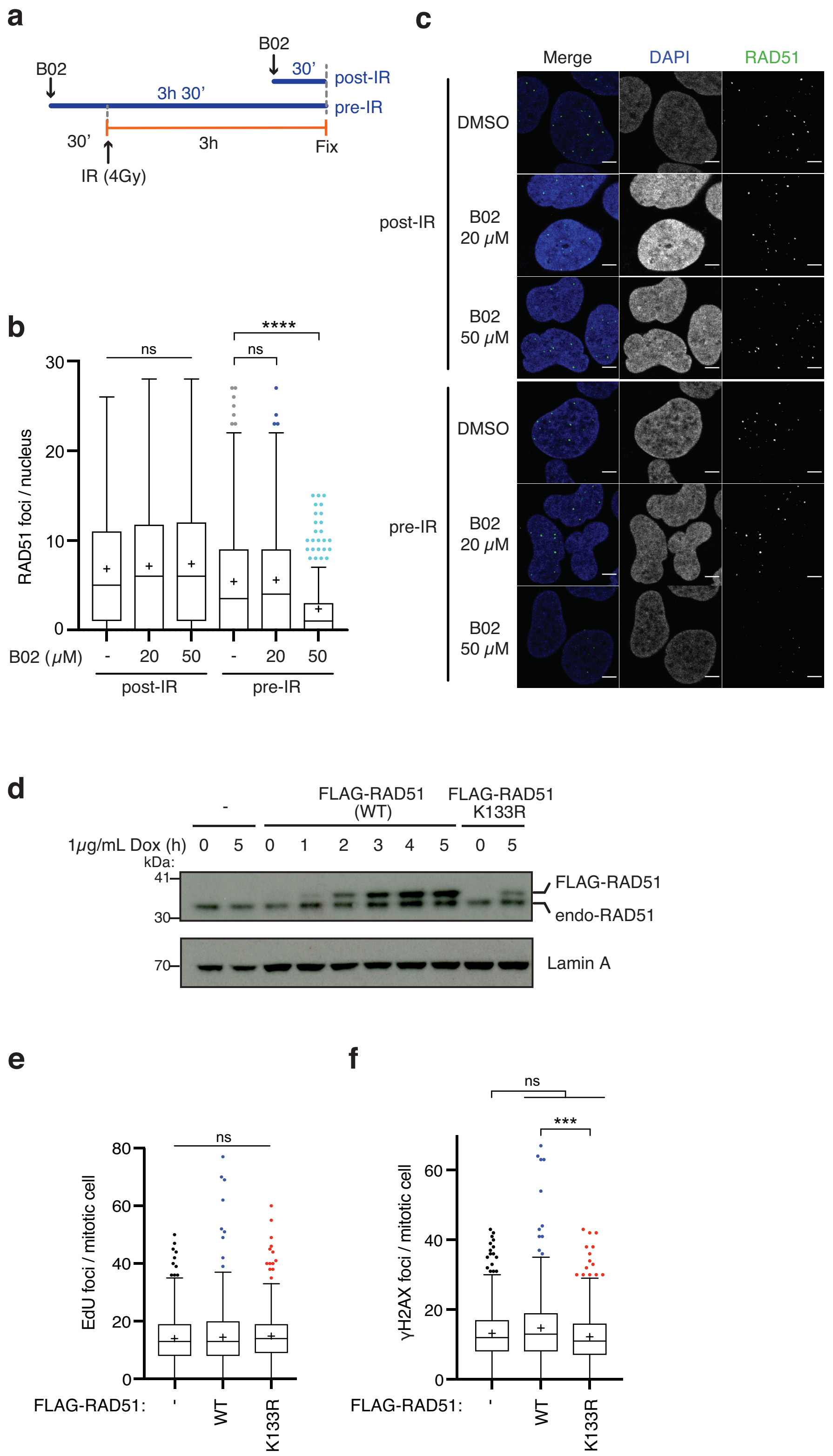

Supplementary Fig. 2: Evaluation of RAD51 inhibitor B02 and conditional expression of RAD51 variants.

a. Schematic diagram of B02 addition before (pre-IR) or after (post-IR) irradiation (4 Gy) of asynchronous U2OS cells. 3 hours after irradiation, cells were fixed and stained for RAD51 IF. **b**. Quantification of the number of RAD51 foci per nucleus. Data were obtained from three independent experiments (100 cells analysed per repeat); n=300 per condition. Data distribution is represented by Tukey box-and-whisker plots. Bounds of box are 25–75th percentile, center shows the median and '+' marks the mean. Whiskers indicate ±1.5xIQR, data

outside this range are drawn as individual dots. *p*-values were calculated by a Kruskal-Wallis test followed by Dunn's multiple comparison test. Asterisks indicate ****p value ≤ 0.0001. **c.** Representative images of cells quantified in (b). Scale bar indicates 5 µm. **d.** Western blot assessing RAD51 and Lamin A (loading control) levels in asynchronous U2OS Flp-In T-REx cells expressing the indicated FLAG-RAD51 variants from the tet-O promoter. Based on a single experiment. **e-f.** Quantification of the number of EdU foci (e) and γ-H2AX foci (f) per mitotic cell in doxycycline treated cells. Data was obtained from four independent experiments; n=400 per condition. Data distribution is represented by Tukey box-and-whisker plots. Bounds of box are 25–75th percentile, center shows the median and '+' marks the mean. Whiskers indicate ±1.5xIQR, data outside this range are drawn as individual dots. *p*-values were calculated by a Kruskal-Wallis test followed by Dunn's multiple comparison test. Asterisks indicate ***p value ≤ 0.001. Source data are provided as a Source Data file.

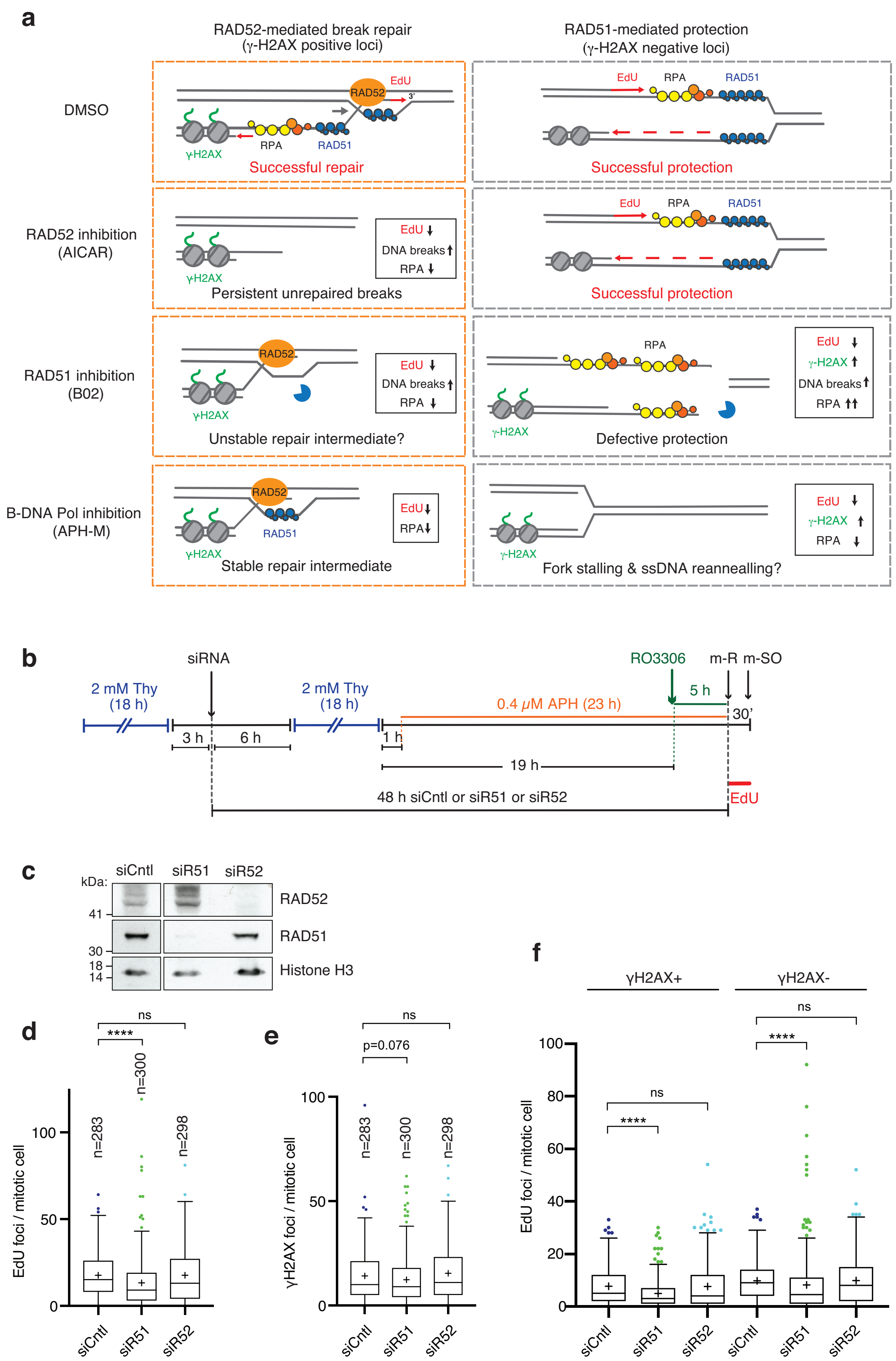

Supplementary Fig. 3: The impact of RAD51 and RAD52 depletion on MiDAS.

a. A model for MiDAS at γH2AX-positive and γH2AX-negative loci. Stalled replication forks are re-activated to complete DNA synthesis in mitosis. A subset of stalled forks is cleaved in mitosis to undergo γH2AX-

positive MiDAS via a RAD52-mediated BIR-like mechanism. RAD51-mediated DNA protection prevents nucleolytic attack of both DNA repair intermediates and continuing replication forks in mitosis, thereby promoting MiDAS at broken and unbroken forks. Upon mitotic RAD52 inhibition (AICAR), broken forks no longer engage in BIR-like MiDAS, resulting in the reduction of γH2AX-positive mitotic EdU foci; decreased mitotic RPA foci formation and persistent mitotic DNA breaks. In the absence of mitotic RAD51 recruitment (B02), RPA-bound ssDNA structures at mitotic DNA repair intermediates and ongoing replication forks are vulnerable to nucleolytic attack, resulting in the reduction of both γH2AX-positive and γH2AX-negative mitotic EdU foci and the increased induction mitotic DNA breaks. The mitotic inhibition of B-family DNA polymerases (APH-M) abolishes MiDAS at both broken and unbroken replication forks, leading to the accumulation of RAD52-dependent DNA intermediates at broken forks (which are undetectable by PFGE) and γH2AX-associated fork stalling at unbroken forks. **b.** Schematic diagram of experimental procedure for U2OS cell synchronisation combined with siRNA transfection, targeting RAD51 (siR51) or RAD52 (siR52). **c.** Following cell synchronisation according to workflow in (b), the total cell population was collected by trypsinization 30 min after m-R and analysed by western blot for the depletion of the corresponding protein. Based on a single experiment. **d-f.** Quantification of the number of EdU foci (d) and γ-H2AX foci (e) per mitotic cell. Quantification of the number of EdU foci that colocalise with γ-H2AX (γ-H2AX$^+$) and the number of EdU foci that do not colocalise with γ-H2AX (γ-H2AX$^-$) per mitotic cell (f). Data were obtained from three independent experiments (approximately 100 cells analysed per repeat); n= the total number of cells quantified per condition as indicated on graph. Data distribution is represented by Tukey box-and-whisker plots. Bounds of box are 25–75th percentile, center shows the median and '+' marks the mean. Whiskers indicate ±1.5xIQR, data outside this range are drawn as individual dots. *p*-values were calculated by a Kruskal-Wallis test followed by Dunn's multiple comparison test. Asterisks indicate ****p value ≤ 0.0001. Source data are provided as a Source Data file.

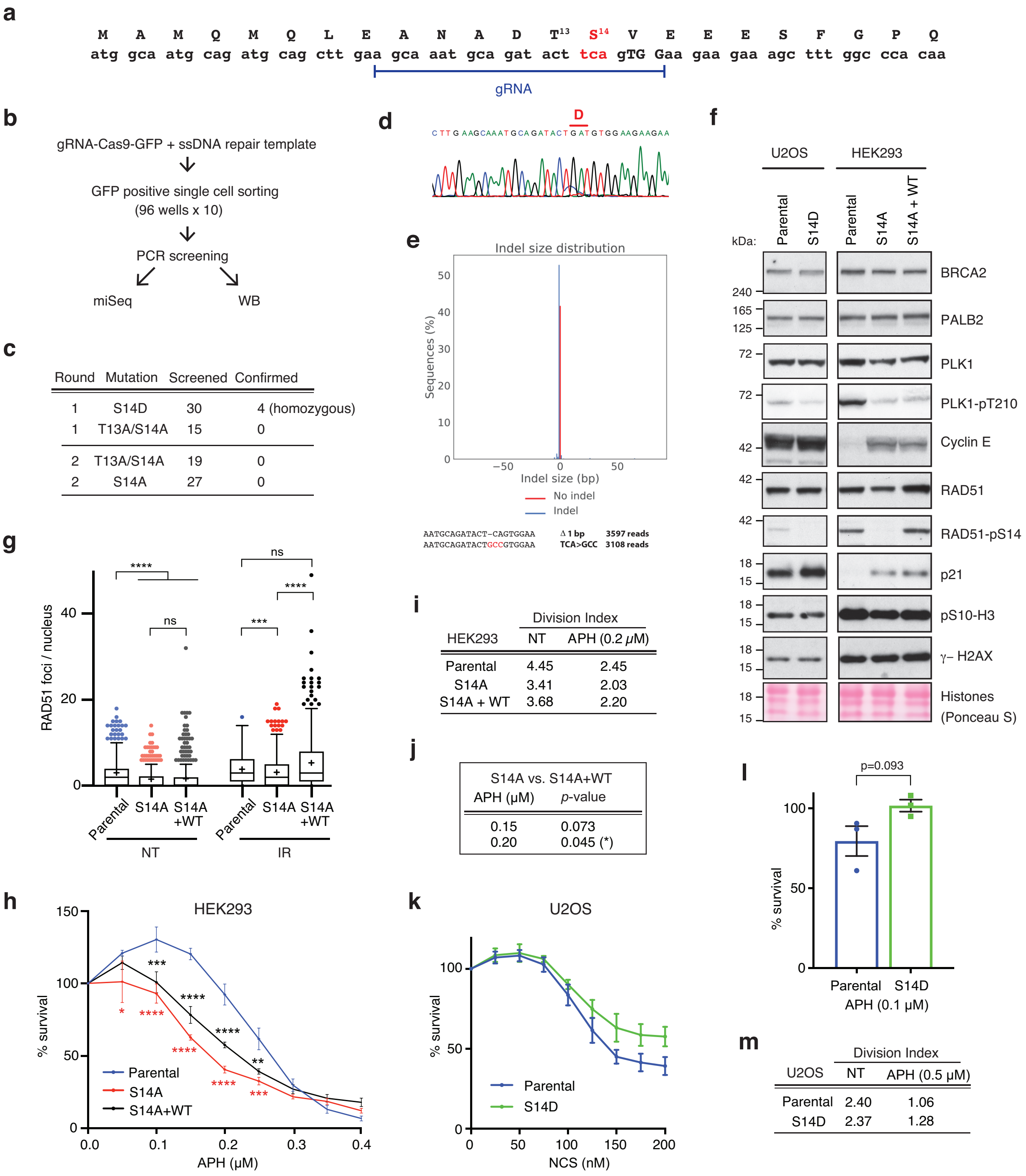

Supplementary Fig. 4: Evaluation of endogenous *RAD51* knock-in mutation at S14.

a. DNA sequence and corresponding amino acid sequence of the *RAD51* S14 locus targeted by gRNA for CRISPR/Cas9-mediated gene editing. **b.** Schematic illustration of workflow for knock-in of *RAD51* S14 missense mutations by CRISPR/Cas9 gene editing. **c.** The total number of CRISPR/Cas9 targeted U2OS clones screened by PCR, and the number of clones validated by Sanger sequencing. Two rounds (Round 1 and 2) of the whole screening procedure, depicted in (b), were attempted. **d.** Representative Sanger sequencing chromatogram of the *RAD51* S14 locus obtained for the U2OS *RAD51*$^{S14D/S14D}$ homozygous knock-in mutant cell lines. **e.** Graphic representation of the indel size distribution for all reads of the *RAD51* S14 locus in the HEK293 knock-in mutant cell line as sequenced by next generation sequencing (miSeq). The corresponding DNA sequences detected are displayed below. **f.** Asynchronous populations of the indicated cell lines were assessed by western blot against S14 phosphorylated RAD51 (RAD51-pS14), RAD51, BRCA2, PALB2, PLK1, phosphorylated PLK1 (PLK1-pT210), Cyclin E, p21, pS10-H3 (mitotic marker) and γ-H2AX. Similar results were obtained

from multiple experiments. **g.** HEK293 cell line was assessed for RAD51 foci in untreated conditions (NT) or 3 hours after irradiation (IR) at 4 Gy. Data were obtained from three independent experiments (150 cells analysed per repeat; n=450 per condition). Data distribution is represented by Tukey box-and-whisker plots. Bounds of box are 25–75th percentile, center shows the median and '+' marks the mean. Whiskers indicate ±1.5xIQR, data outside this range are drawn as individual dots. *p*-values were calculated by a Kruskal-Wallis test followed by Dunn's multiple comparison test. Asterisks indicate ****p* value ≤ 0.001; *****p* value ≤ 0.0001. **h.** Cell survival of HEK293 cell line upon exposure to aphidicolin (APH) was assessed by WST-1 assay. Data are presented as mean values, error bars represent SEM; data were obtained from three independent experiments. *p*-values were calculated by a two-way ANOVA followed by Tukey's multiple comparisons test. Asterisks indicate significant differences compared to the parental HEK293 cell line: **p* value ≤ 0.05; ***p* value ≤ 0.01; ****p* value ≤ 0.001; *****p* value ≤ 0.0001. **i.** Proliferation of the CRISPR gene-edited $RAD51^{S14A/S14A}$ mutant cell line (S14A), the WT RAD51 complemented cell line (S14A+WT) and the corresponding HEK293 parental cell line (Parental) was assessed following 96-hour incubation with 0.5 μM aphidicolin (APH) or non-treated control (NT). The division index for each cell line was determined by Cell Trace™ cell proliferation assay based on a single experiment. **j.** Statistical differences of cell survival between the $RAD51^{S14A/S14A}$ (S14) and the WT RAD51 complemented (S14A+WT) cell lines, as assayed in (h). *p*-values were calculated by a two-way ANOVA followed by Tukey's multiple comparisons test and summarised. **k.** Cell survival upon exposure to neocarzinostatin (NCS) was assessed by WST-1 assay. Data are presented as mean values, error bars represent SEM. Data were obtained from three independent experiments. **l.** Cell survival upon exposure to 0.1 μM APH was assessed by clonogenic assay. Data are presented as mean values, error bars represent SEM. Data were obtained from three independent experiments. *p*-value was calculated by a two-sided unpaired t test. **m.** Proliferation of the CRISPR gene-edited $RAD51^{S14D/S14D}$ mutant cell line (S14D) and the corresponding U2OS parental cell line was assessed following 96 hour incubation with 0.5 μM aphidicolin (APH) or non-treated control (NT). The division index for each cell line was determined by Cell Trace™ cell proliferation assay based on a single experiment. Source data are provided as a Source Data file.

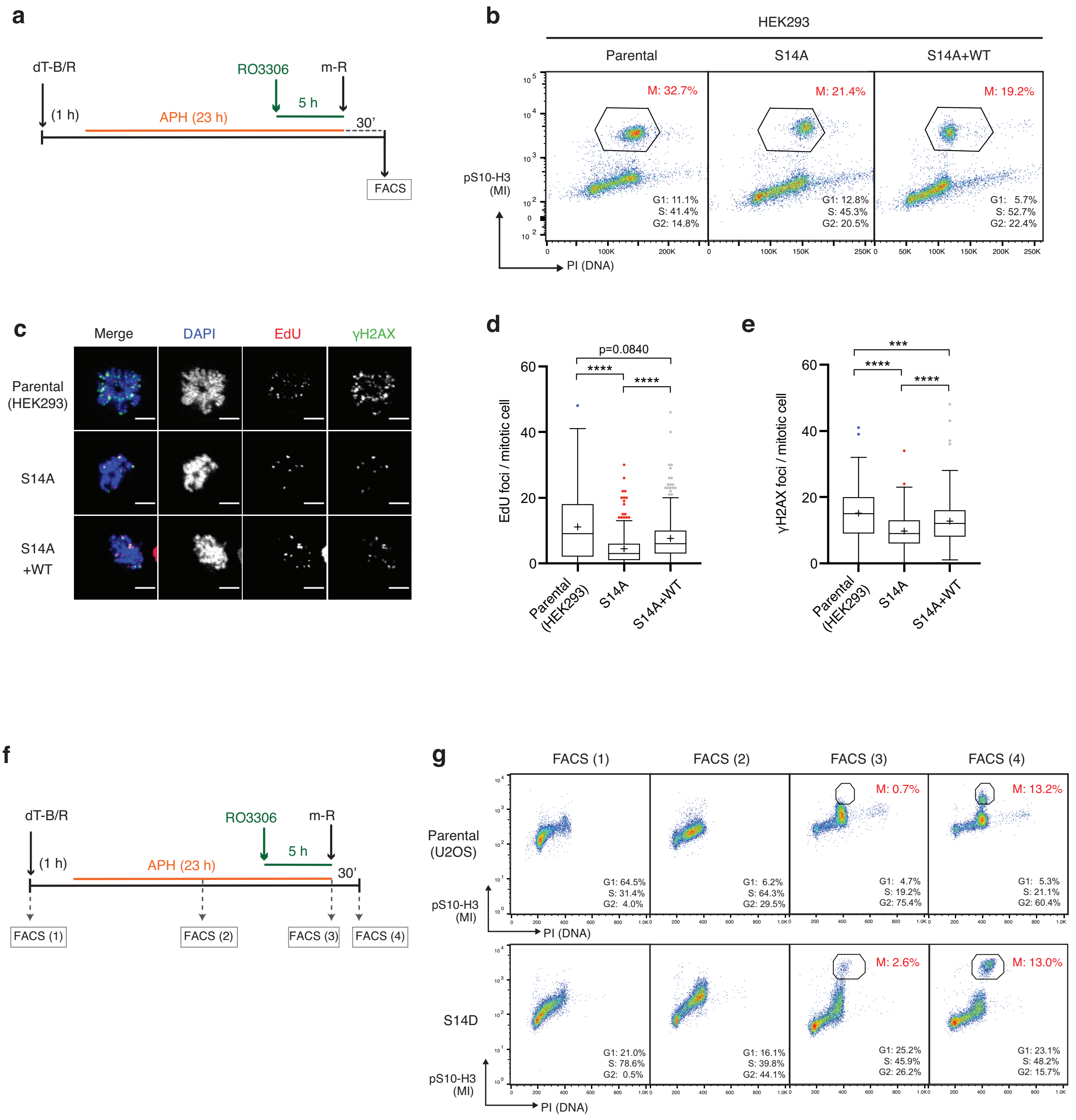

Supplementary Fig. 5: Synchronization of *RAD51* S14 knock-in mutant cell lines.
a. Schematic diagram of experimental procedure used to obtain data in (b). The total cell population was collected and analysed by FACS at m-R. **b.** FACS analysis of mitotic index (pS10-H3) and DNA content (PI) in the CRISPR gene-edited HEK293 cell lines following cell synchronization according to the experimental procedure depicted in (a). The G1, S and G2 populations were estimated by the Watson Pragmatic algorithm (FlowJo), based on the PI staining of interphase cells. **c-e.** EdU incorporation and γ-H2AX IF signal were analysed in the parental HEK293 cell line; the CRISPR gene-edited $RAD51^{S14A/-}$ mutant cell line (S14A) and the wild-type RAD51 complemented $RAD51^{S14A/-}$ cell line (S14A+WT) following cell synchronization as depicted in Fig. 4b. Representative images of the data analysed (c). Scale bar indicates 5 μm. Quantification of the number of EdU foci (d) and the number of γ-H2AX foci per mitotic cell (e). Data were obtained from three independent experiments (100 cells analysed per repeat); n=300 per condition. Data distribution are represented by Tukey box-and-whisker plots. Bounds of box are 25–75th percentile, center shows the median and ‘+’ marks the mean. Whiskers indicate ±1.5xIQR, data outside this range are drawn as individual dots. *p*-values were calculated by a Kruskal-Wallis test followed by Dunn’s multiple comparison test. Asterisks indicate ***p value ≤ 0.001; ****p value ≤ 0.0001. **f.** Schematic diagram of experimental procedure used to obtain data in (d). The total cell population was collected and analysed by FACS at the indicated timepoints. **g.** FACS analysis of

mitotic index (pS10-H3) and DNA content (PI) in the U2OS parental cell line and CRISPR gene-edited *RAD51*$^{S14D/S14D}$ mutant cell line (S14D) following cell synchronization according to the experimental procedure depicted in (f). Source data are provided as a Source Data file.

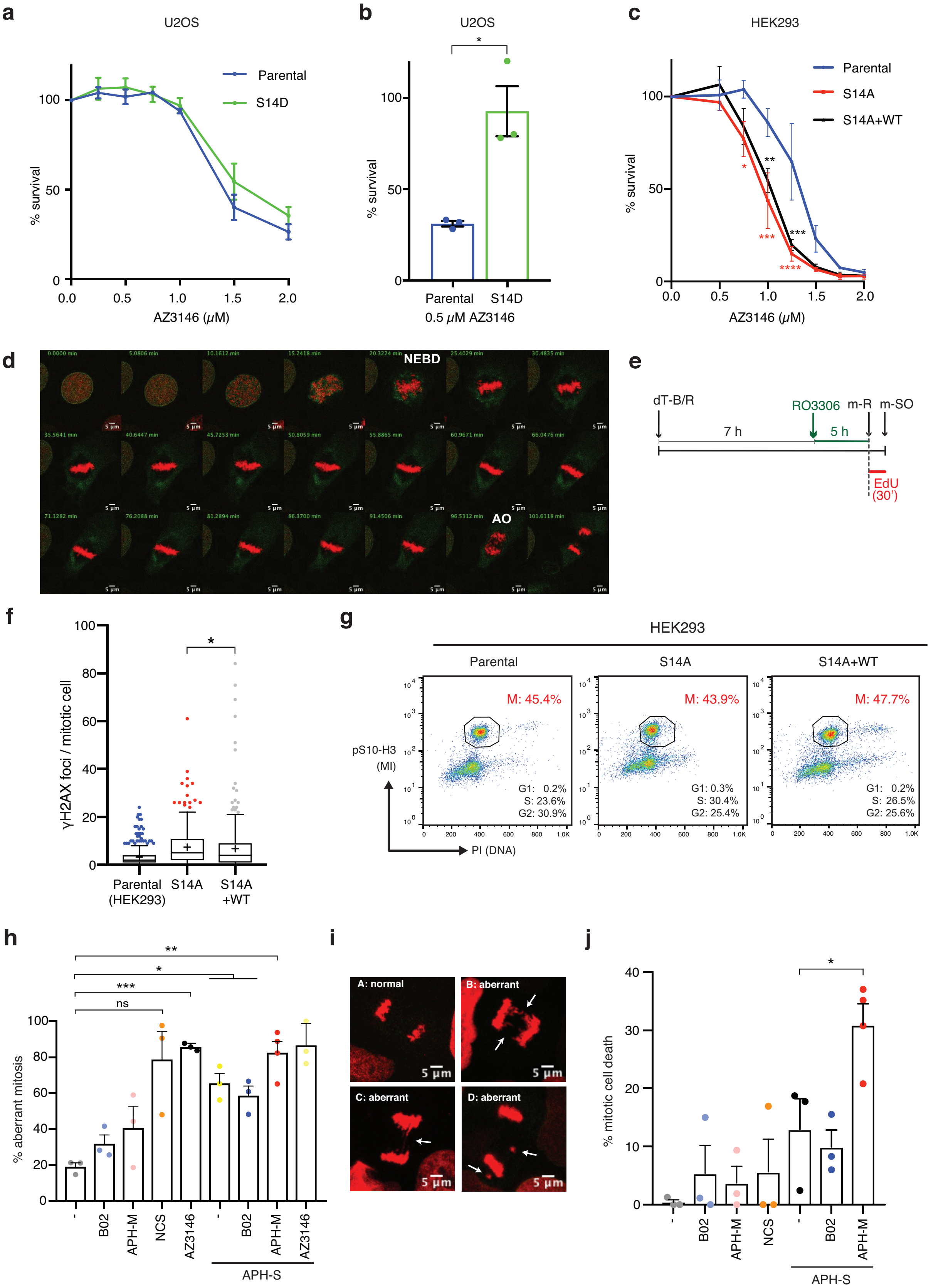

Supplementary Fig. 6: Impact of RAD51 and MiDAS on mitotic progression.

a. Cell survival of U2OS parental cell line and U2OS *RAD51*$^{S14D/S14D}$ knock-in mutant cell line (S14D) upon exposure to AZ3146, as determined by WST-1 assay. Data are presented as mean values, error bars represent SEM. Data were obtained from four independent experiments. **b**. Clonogenic cell survival of U2OS parental

wild-type and U2OS *RAD51*$^{S14D/S14D}$ knock-in mutant cell line (S14D) upon exposure to 0.5 μM AZ3146. Data are presented as mean values, error bars represent SEM. Data were obtained from three independent experiments. The significant difference between the samples was assessed by a two-sided unpaired Welch's t test. Asterisks indicate **p* value ≤ 0.05. **c**. Survival of HEK293 cell lines upon exposure to MPS1 inhibitor (AZ3146), as determined by WST-1 assay. Data are presented as mean values, error bars represent SEM. Data were obtained from three independent experiments. Significant differences compared to the parental HEK293 cell line were determined by a two-way ANOVA followed by Tukey's multiple comparisons test. Asterisks indicate **p* value ≤ 0.05; ***p* value ≤ 0.01; ****p* value ≤ 0.001; *****p* value ≤ 0.0001. **d.** Representative image of mitotic progression analysed in U2OS cells following cell synchronisation as in Fig. 5d. Frames displaying nuclear envelope breakdown (NEBD) and anaphase onset (AO) are indicated. **e.** Schematic diagram of cell synchronisation for HEK293 cell lines used to obtain data in (f). **f.** Quantification of the number of γ-H2AX foci per mitotic cell, detected in mitotic cells collected by m-SO. Data were obtained from three independent experiments (100 cells analysed per repeat); n=300 per cell line. *p* value was calculated by a Kruskal-Wallis test followed by Dunn's multiple comparison test. Asterisk indicates **p* value ≤ 0.05. **g.** Cells were synchronized according following the protocol depicted in (e). The total cell population was collected at 30 min after m-R and analysed by FACS for mitotic index (pS10-H3) and DNA content (PI). The G1, S and G2 populations in respective cell lines were estimated by the Watson Pragmatic algorithm (FlowJo), based on the PI staining of interphase cells. **h.** Percentage of mitotic aberrations (lagging chromosomes, anaphase bridges) was quantified for data analysed in Fig. 5d. Data are presented as mean values, error bars represent SEM. Data were obtained from at least three independent experiments. *p* values were calculated by a Brown-Forsythe and Welch ANOVA test followed by Dunnett's T3 multiple comparisons test. Asterisks indicate **p* value ≤ 0.05; ***p* value ≤ 0.01; ****p* value ≤ 0.001. **i.** Representative images of normal (A) and aberrant mitotic chromatin (B-D) as quantified in (h). Arrows indicate lagging chromosomes, chromatin bridges or micronuclei. **j.** The percentage of cells that die before completing mitosis was quantified for data analysed in Fig. 5d. Data are presented as mean values, error bars represent SEM. Data were obtained from at least three independent experiments. *p* values were calculated by a Brown-Forsythe and Welch ANOVA test followed by Dunnett's T3 multiple comparisons test. Asterisk indicates **p* value ≤ 0.05. Source data are provided as a Source Data file.

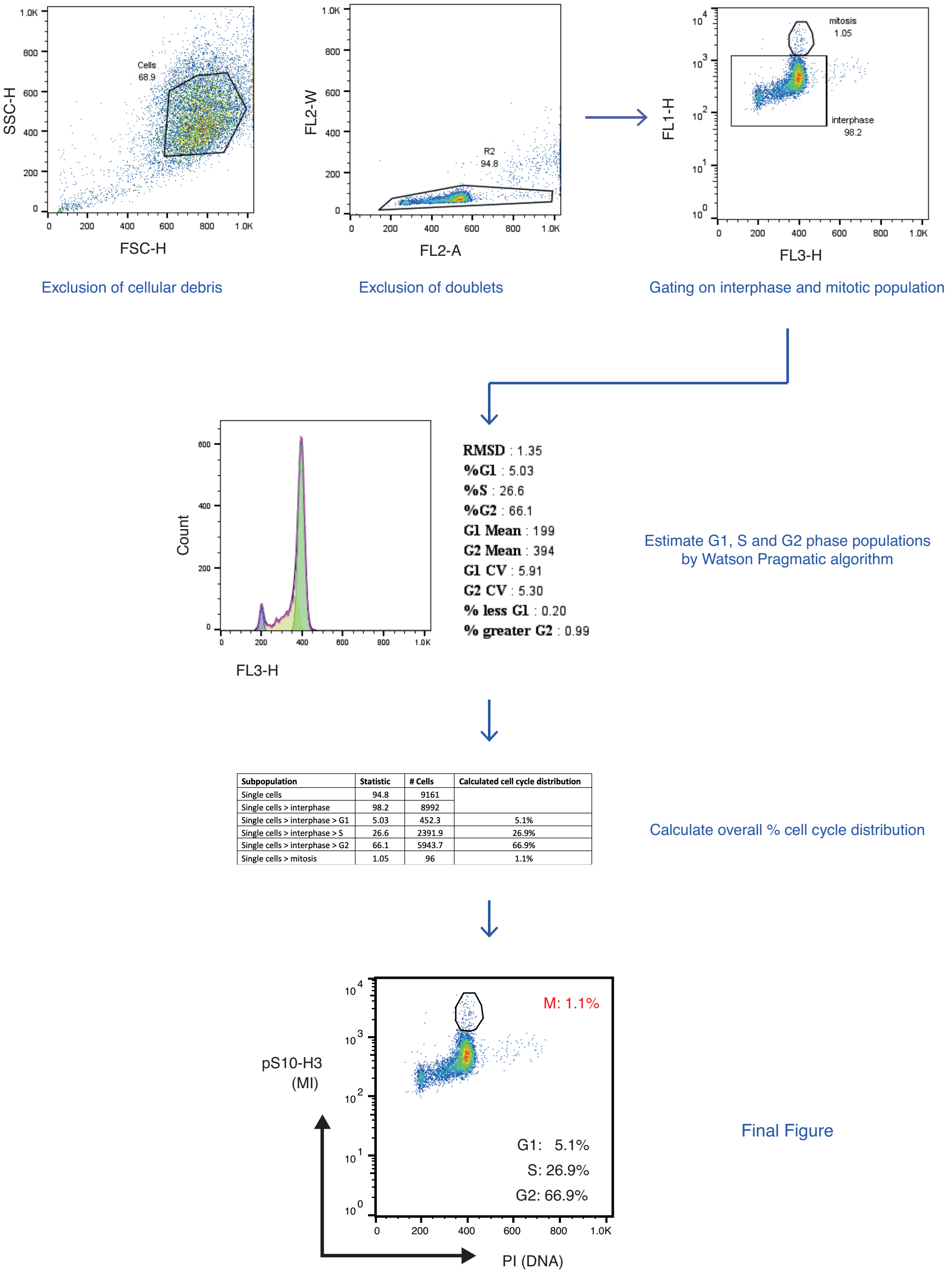

Subpopulation	Statistic	# Cells	Calculated cell cycle distribution
Single cells	94.8	9161	
Single cells > interphase	98.2	8992	
Single cells > interphase > G1	5.03	452.3	5.1%
Single cells > interphase > S	26.6	2391.9	26.9%
Single cells > interphase > G2	66.1	5943.7	66.9%
Single cells > mitosis	1.05	96	1.1%

Supplementary Fig. 7: **Gating strategy.**

Forward versus side scatter (FSC vs. SSC) gating was used to remove debris from the main cell population where necessary; single cells were discriminated from doublets by FL2A vs. FL2W gating. Histone H3 S10 phosphorylation staining intensity (mitotic cells) is measured on a logarithmic scale on the Y-axis while PI staining on the X-axis measures the doubling of DNA content (i.e. 2C to 4C) to identify interphase and mitotic cell populations. Cell counts in G1, S, G2 were estimated using the Watson Pragmatic algorithm in FlowJo on gated interphase cells and combined with quantification of gated mitotic cells to calculate cell cycle distributions.

Supplementary Table 1: ssDNA template used to edit *RAD51* gene by CRISPR/Cas9

name	sequence
S14D	5'-CCAAAATTAGCAACTAACCACATACCTCTAACCGTGAAATGGGTTGTGGGCC AAAGCTTTCTTCTTCCAC**ATC**AGTATCTGCATTTGCTTCAAGCTGCATCTGCAT TGCCATTACTGAAAAATACAAATGCTTATCAGTATAAACACTAG-3'
S14A	5'-CCAAAATTAGCAACTAACCACATACCTCTAACCGTGAAATGGGTTGTGGGCC AAAGCTTTCTTCTTCCAC**GGC**AGTATCTGCATTTGCTTCAAGCTGCATCTGCAT TGCCATTACTGAAAAATACAAATGCTTATCAGTATAAACACTAG-3'

Supplementary Table 2: Primers used to screen *RAD51* knock-in mutation at S14

name	sequence
WT reverse	5'-CTTGAAGCAAATGCAGATACT**TCA**-3'
S14A reverse	5'-CTTGAAGCAAATGCAGATACT**GCC**-3'
S14D reverse	5'-CTTGAAGCAAATGCAGATACT**GAT**-3'
forward	5'-TGCAGCCTTTCCACAGGATT-3'

Supplementary Methods

CRISPR/Cas9-mediated editing of *RAD51* gene

Cells were co-transfected with (i) a plasmid carrying Cas9, GFP and a guide RNA targeting *RAD51* S14 locus (AGCAAATGCAGATACTTCAGTGG) and (ii) the relevant ssDNA template (Supplementary Table 1), using Lipofectamin LTX with Plus Reagent (ThermoFisher Scientific) according to manufacturer's instructions. At 24 hours after transfection, GFP-expressing cells were sorted by FACS (MoFlo XDP, Beckman Coulter). Single clones were expanded and genomic DNA was extracted for PCR amplification of the targeted *RAD51* S14 locus. Clones were screened for the desired mutation using forward primers that are perfectly complementary to either the wildtype S14 sequence or the mutated S14D or S14A sequence (Supplementary Table 2). Clones that generated a convincing PCR product with the mutation-specific primer, but not the wild-type primer, were further verified for the knock-in mutation by Sanger sequencing. For heterozygous clones, next-generation sequencing (Illumina MiSeq Next Generation Sequencer) was used to determine the allele-specific sequences at the *RAD51* S14 locus. Verified clones were further tested for the absence of RAD51 phosphorylation by western blot.

Antibodies

Primary antibodies (supplier name, catalog number) and dilutions for western blot (WB), immunofluorescence (IF) and flow cytometry (FC) used in this study are: anti-phospho-Histone H2A.X (Ser139), clone JBW301 (Merck Millipore, 05-636, WB 1:1000, IF 1:1000); anti-Histone H3 (Bethyl Laboratories, A300-822A, WB 1:1000); anti-phospho-Histone H3 (Ser 10) (Merck Millipore, 06-570, WB 1:1000, FC 1:100); anti-PLK1 (Bethyl Laboratories, A300-251A, WB 1:1000); anti-phospho-PLK1 (T210) (BD Biosciences, 558400, WB 1:1000); anti-BRCA2 (Sigma-Aldrich, OP95, WB 1:1000); anti-PALB2 (Biorbyt, orb412704, WB 1:1000); anti-cyclin E (Santa Cruz Biotechnology, HE12, WB 1:1000); anti-phospho-RAD51 S14 (generated as described in Yata et al. 2012[1], WB 1:1000); anti-p21 (Cell Signalling, 12D1, WB 1:1000); anti-RAD52 (Santa Cruz, sc-365341, WB 1:200); anti-RAD51 (7946, generated as described in Yata et al. 2014[2], WB 1:5000, IF 1:1000); anti-RPA (Bethyl Laboratories, A300-244A, IF 1:5000). Secondary antibodies used are: anti-rabbit HRP (Daco, P0448, WB 1:2000); anti-mouse HRP (Daco, P0447, WB 1:1000); anti-rabbit Alexa Fluor 488 (Thermo Fisher Scientific, A-11008, IF 1:500, FC 1:100); anti-mouse Alexa Fluor 555 (Thermo Fisher Scientific, A-21422, IF 1:500, FC 1:100); and anti-rabbit Alexa 647 (Thermo Fisher Scientific, A-21244, IF 1:500, FC 1:100). All antibodies were validated by suppliers and prior publications, and show the band of the expected size.

Cell Trace™ Cell proliferation assay

Cells were stained following the manufacturer's protocol (Cell Trace™ Cell Proliferation kit, ThermoFisher Scientific, C34564). $4x10^6$ cells were incubated in 0.5 µM Cell Trace Far red solution at a cell density of 10^6 per ml at 37 °C for 20 min in PBS. Four volumes of complete medium were added, and cells were incubated for 5 min at 37 °C to absorb unbound dye. Unbound dye was removed, and cells were seeded in fresh complete medium at a density of 300 000 cells for U2OS and 500 000 cells for HEK293 per 10-cm dish. Cells were incubated in aphidicolin at the indicated concentrations or left untreated. Untreated cells were harvested by trypsinization at 24 hour after seeding to determine the undivided population. Untreated and aphidicolin-treated samples were harvested by trypsinization at 96 hour after seeding to assess the effect of aphidicolin on proliferation.

Cells were stained with DAPI (50 ng/ml) to distinguish live and dead cells. Cells were analysed using a Cytek DxP8 Flow cytometer equipped with FlowJo CE software (version 7.5.110.7). The obtained proliferation curve was fitted using FlowJo analysis software (version 10.5.3). Parameters were fixed at 0.5 Peak Ratio and Peak was defined CV based on the undivided population as analysed at 24h.

CO-FISH

Metaphase spreads were rehydrated in PBS before being treated with 0.5 mg/ml RNAse A (Sigma-Aldrich) in PBS for 10 min at 37 ^{o}C. The spreads were next stained with 0.5 µg/ml Hoechst 33258 (Sigma-Aldrich) in 2x SSC for 15 min at room temperature and exposed to 6500 J/m^2 365 nm UV light. To degrade the BrdU:BrdC-containing DNA strands, the metaphase spreads were incubated with 10 U/µl Exonuclease III (Promega) in the manufacturer's buffer at 37 ^{o}C for 30 min. The spreads were dehydrated by consecutive incubations in 70%, 90% and 100% ethanol and air-dried. The spreads were next incubated with the PNA probe (F3004, PNABio) in hybridising solution (10 mM Tris-HCl pH 7.2, 70% formamide and 0.5% w/v blocking reagent (Roche)) for 2 hours. The metaphase spreads were briefly rinsed in hybridisation wash 1 (10 mM Tris-HCl pH7.2, 70% formamide and 0.1% BSA) and incubated with the complementary PNA probe (F3009, PNABio) in hybridising solution. The metaphase spreads were washed in hybridisation wash 1 for 15 min, followed by three 5 min washes in hybridisation wash 2 (0.1 M Tris-HCl pH 7.2, 0.15 M NaCl, 0.08% Tween-20). DNA was counterstained by adding 0.7 µg/ml DAPI (Sigma Aldrich) to the second wash. The spreads were dehydrated by consecutive incubations in 70%, 90% and 100% ethanol and air-dried, then finally mounted in ProLong Gold antifade reagent (Life Technologies).

Supplementary Reference

1 Yata, K. *et al.* Plk1 and CK2 act in concert to regulate Rad51 during DNA double strand break repair. *Mol Cell* **45**, 371-383 (2012).

2 Yata, K. *et al.* BRCA2 coordinates the activities of cell-cycle kinases to promote genome stability. *Cell Rep* **7**, 1547-1559 (2014).
