# Supplementary material for: The RAD51 recombinase protects mitotic chromatin in human cells
Source: Nat Commun. 2021 Sep 10;12:5380. doi: 10.1038/s41467-021-25643-y (PMC8433380; doi:10.1038/s41467-021-25643-y)
Supplement: Supplementary file 3 — Description of Additional Supplementary Files [file 41467_2021_25643_MOESM3_ESM.pdf]

Description of Additional Supplementary Files

Supplementary Movie 1: Representative mitotic progression of HEK293 parental cell. Following synchronisation as depicted in Fig. 5a.

Supplementary Movie 2: Representative mitotic progression of HEK293 S14A cell. Following synchronisation as depicted in Fig. 5a.

Supplementary Movie 3: Representative mitotic progression of HEK293 S14A+WT cell. Following synchronisation as depicted in Fig. 5a.

Supplementary Movie 4: Representative mitotic progression of U2OS cell. Following synchronisation without aphidicolin (APH) exposure in S phase (-), in the presence of DMSO vehicle as depicted in Fig. 5c.

Supplementary Movie 5: Representative mitotic progression of U2OS cell. Following synchronisation with aphidicolin (APH) exposure in S phase (APH-S) as depicted in Fig. 5c.

Supplementary Movie 6: Representative mitotic progression of U2OS cell in the presence of 20 µM B02. Following synchronisation without aphidicolin (APH) exposure in S phase (-) as depicted in Fig. 5c.

Supplementary Movie 7: Representative mitotic progression of U2OS cell in the presence of 20 µM B02. Following synchronisation with aphidicolin (APH) exposure in S phase (APH-S) as depicted in Fig. 5c.

Supplementary Movie 8: Representative mitotic progression of U2OS cell in the presence of 2 µM aphidicolin (APH-M). Following synchronisation without aphidicolin (APH) exposure in S phase (-) as depicted in Fig. 5c.

Supplementary Movie 9: Representative mitotic progression of U2OS cell in the presence of 2 µM aphidicolin (APH-M). Following synchronisation with aphidicolin (APH) exposure in S phase (APH-S) as depicted in Fig. 5c.

Supplementary Movie 10: Representative mitotic progression of U2OS cell in the presence of 2 µM AZ3146. Following synchronisation without aphidicolin (APH) exposure in S phase (-) as depicted in Fig. 5c.

Supplementary Movie 11: Representative mitotic progression of U2OS cell in the presence of 2 µM AZ3146. Following synchronisation with aphidicolin (APH) exposure in S phase (APH-S) as depicted in Fig. 5c.

Supplementary Movie 12: Representative mitotic progression of U2OS cell in the presence of 50 nM neocarzinostatin (NCS). Following synchronisation in the absence of aphidicolin as depicted in Fig. 5c.
