# Supplementary material for: The RAD51 recombinase protects mitotic chromatin in human cells
Source: Nat Commun. 2021 Sep 10;12:5380. doi: 10.1038/s41467-021-25643-y (PMC8433380; doi:10.1038/s41467-021-25643-y)
Supplement: Supplementary file 16 — Reporting Summary [file 41467_2021_25643_MOESM16_ESM.pdf]

nature research

Corresponding author(s): Fumiko Esashi

Last updated by author(s): Aug 3, 2021

Reporting Summary

Nature Research wishes to improve the reproducibility of the work that we publish. This form provides structure for consistency and transparency in reporting. For further information on Nature Research policies, see our Editorial Policies and the Editorial Policy Checklist.

Statistics

For all statistical analyses, confirm that the following items are present in the figure legend, table legend, main text, or Methods section.

n/a	Confirmed	
☐	☒	The exact sample size (*n*) for each experimental group/condition, given as a discrete number and unit of measurement
☐	☒	A statement on whether measurements were taken from distinct samples or whether the same sample was measured repeatedly
☐	☒	The statistical test(s) used AND whether they are one- or two-sided<br>*Only common tests should be described solely by name; describe more complex techniques in the Methods section.*
☒	☐	A description of all covariates tested
☐	☒	A description of any assumptions or corrections, such as tests of normality and adjustment for multiple comparisons
☐	☒	A full description of the statistical parameters including central tendency (e.g. means) or other basic estimates (e.g. regression coefficient) AND variation (e.g. standard deviation) or associated estimates of uncertainty (e.g. confidence intervals)
☐	☒	For null hypothesis testing, the test statistic (e.g. *F*, *t*, *r*) with confidence intervals, effect sizes, degrees of freedom and *P* value noted<br>*Give P values as exact values whenever suitable.*
☒	☐	For Bayesian analysis, information on the choice of priors and Markov chain Monte Carlo settings
☒	☐	For hierarchical and complex designs, identification of the appropriate level for tests and full reporting of outcomes
☒	☐	Estimates of effect sizes (e.g. Cohen's *d*, Pearson's *r*), indicating how they were calculated

Our web collection on statistics for biologists contains articles on many of the points above.

Software and code

Policy information about availability of computer code

Data collection	For microscopy analysis of fixed samples, all images were captured using an Olympus FV1000 Laser Scanning Microscope with Becker and Hickel FLIM system and Olympus Fluoview FV1200 using FV-ASW software (version 4.2). Live cell images were captured using a Zeiss 880 inverted confocal microscope with Zen Black software (version 14.0.22.201). Flow cytometry for cell cycle analyses was performed on FACSCalibur (Becton Dickinson) equipped with CellQuest Pro software (version 6.0) or CytoFLEX LX (Becton Dickinson) equipped with the CytExpert programme (version 2.3.0.84). Cell Trace Cell proliferation assay was conducted using Cytek DxP8 Flow cytometer equipped with FlowJo CE software (version 7.5.110.7).
Data analysis	Foci counting was performed using FIJI (Image J version 2.0.0-rc-65/1.52a or 2.1.0) and the GDSC (FindFoci) ImageJ plugin (ref 60). Cell cycle distribution was assessed using FlowJo software (versions 10.5.3, 10.6.2 or 10.7.2). Cell proliferation was assessed using FlowJo software (versions 10.5.3)<br>Ref 60: Herbert AD, Carr AM, Hoffmann E (2014) FindFoci: A Focus Detection Algorithm with Automated Parameter Training That Closely Matches Human Assignments, Reduces Human Inconsistencies and Increases Speed of Analysis. PLoS ONE 9(12): e114749.

For manuscripts utilizing custom algorithms or software that are central to the research but not yet described in published literature, software must be made available to editors and reviewers. We strongly encourage code deposition in a community repository (e.g. GitHub). See the Nature Research guidelines for submitting code & software for further information.

Data

Policy information about availability of data

All manuscripts must include a data availability statement. This statement should provide the following information, where applicable:

- Accession codes, unique identifiers, or web links for publicly available datasets
- A list of figures that have associated raw data
- A description of any restrictions on data availability

The source data generated and analyzed in this study are available in the Open Science Framework (OSF) with the identifier [doi:10.17605/OSF.IO/68X3K] (Ref 61)

Field-specific reporting

Please select the one below that is the best fit for your research. If you are not sure, read the appropriate sections before making your selection.

[x] Life sciences [ ] Behavioural & social sciences [ ] Ecological, evolutionary & environmental sciences

For a reference copy of the document with all sections, see nature.com/documents/nr-reporting-summary-flat.pdf

Life sciences study design

All studies must disclose on these points even when the disclosure is negative.

Sample size	No statistical methods were used to predetermine sample size. Sample size was estimated on the basis of similar research reported in the literature (e.g. Mason, Jennifer M., et al. Nature communications 10.1 (2019): 1-11). Foci analysis was performed with at least n=3 independent biological replicates, with 100 cells imaged per replicate to establish a high degree of statistical significance.
Data exclusions	No data were excluded from analysis.
Replication	The number of biological replicates is indicated and reproduced the representative data shown in the figures.
Randomization	When acquiring images investigators were blind to fluorescence channels other than DAPI. This facilitated specific imaging of mitotic cells (selected based on the DAPI staining) while preventing biased selection based on the other fluorescence signals to be analysed.
Blinding	Blinding was not relevant when analysing data with software, as the same parameters were used across the compared samples. When analysing data by eye, investigators were blinded.

Reporting for specific materials, systems and methods

We require information from authors about some types of materials, experimental systems and methods used in many studies. Here, indicate whether each material, system or method listed is relevant to your study. If you are not sure if a list item applies to your research, read the appropriate section before selecting a response.

Materials & experimental systems

n/a	Involved in the study
[ ]	[x] Antibodies
[ ]	[x] Eukaryotic cell lines
[x]	[ ] Palaeontology and archaeology
[x]	[ ] Animals and other organisms
[x]	[ ] Human research participants
[x]	[ ] Clinical data
[x]	[ ] Dual use research of concern

Methods

n/a	Involved in the study
[x]	[ ] ChIP-seq
[ ]	[x] Flow cytometry
[x]	[ ] MRI-based neuroimaging

Antibodies

Antibodies used

Primary antibodies (supplier name, catalog number) and dilutions for western blot (WB), immunofluorescence (IF) and flow cytometry (FC) used in this study are:
anti-phospho-Histone H2A.X (Ser139), clone JBW301 (Merck Millipore, 05-636, WB 1:1000, IF 1:1000)
anti-Histone H3 (Bethyl Laboratories, A300-822A, WB 1:1000)
anti-phospho-Histone H3 (Ser 10) (Merck Millipore, 06-570, WB 1:1000, FC 1:100)
anti-PLK1 (Bethyl Laboratories, A300-251A, WB 1:1000)
anti-phospho-PLK1 (T210) (BD Biosciences, 558400, WB 1:1000)
anti-BRCA2 (Sigma-Aldrich, OP95, WB 1:1000)
anti-PALB2 (Biorbyt, orb412704, WB 1:1000)

anti-cyclin E (Santa Cruz Biotechnology, HE12, WB 1:1000)
anti-phospho-RAD51 S14 (generated as described in Yata et al. 2012, WB 1:1000)
anti-p21 (Cell Signalling, 12D1, WB 1:1000);
anti-RAD52 (Santa Cruz, sc-365341, WB 1:200)
anti-RAD51 (7946, generated as described in Yata et al. 2014, WB 1:5000, IF 1:1000)
anti-RPA (Bethyl Laboratories, A300-244A, IF 1:5000).

Secondary antibodies used (supplier name, catalog number) and dilutions for western blot (WB), immunofluorescence (IF) and flow cytometry (FC) are:
anti-rabbit HRP (Daco, P0448, WB 1:2000)
anti-mouse HRP (Daco, P0447, WB 1:1000)
anti-rabbit Alexa Fluor 488 (Thermo Fisher Scientific, A-11008, IF 1:500, FC 1:100)
anti-mouse Alexa Fluor 555 (Thermo Fisher Scientific, A-21422, IF 1:500, FC 1:100)
and anti-rabbit Alexa 647 (Thermo Fisher Scientific, A-21244, IF 1:500, FC 1:100)

Validation

All antibodies used in this study, except these against phospho-RAD51 S14 and RAD51, are commercially available (see catalog numbers above), validated by suppliers and prior publications, and show the band of the expected size. Where relevant, the reduced signals upon siRNA treated cells were also confirmed in this study (Fig 1b for RAD51 and Supplementary Fig 3c for RAD52). The antibody against phospho-RAD51 S14 has been thoroughly validated and used in Yata et al., 2012 and Yata et al., 2014. The antibody against RAD51 has been validated and used in various publications from the group, to include Yata et al., 2012, Yata et al., 2014, Bleuyard et al., 2012, and has been widely shared with other groups for their independent studies, to include Moudry et al., 2016.

Moudry P, Watanabe K, Wolanin KM, Bartkova J, Wassing IE, Watanabe S, Strauss R, Troelsgaard Pedersen R, Oestergaard VH, Lisby M, Andujar-Sanchez M, Maya-Mendoza A, Esashi F, Lukas J, Bartek J. (2016) TOPBP1 regulates RAD51 phosphorylation and chromatin loading and determines PARP inhibitor sensitivity. J Cell Biol. 212:281-8.
Yata K, Bleuyard JY, Nakato R, Ralf C, Katou Y, Schwab RA, Niedzwiedz W, Shirahige K, Esashi F. (2014) BRCA2 coordinates the activities of cell-cycle kinases to promote genome stability. Cell Rep. 7:1547-1559.
Yata K, Lloyd J, Maslen S, Bleuyard JY, Skehel M, Smerdon SJ, Esashi F. (2012) Plk1 and CK2 act in concert to regulate Rad51 during DNA double strand break repair. Mol Cell. 45:371-83.

Eukaryotic cell lines

Policy information about cell lines

Cell line source(s)	U2OS and HEK293 cell lines were acquired from ATCC. U2OS Flp-In T-REx cell line was a kind gift from Dr Daniel Durocher
Authentication	All cell lines authenticated by STR method.
Mycoplasma contamination	All cell lines tested negative for mycoplasma contamination.
Commonly misidentified lines (See ICLAC register)	No commonly misidentified cell lines were used.

Flow Cytometry

Plots

Confirm that:

- [x] The axis labels state the marker and fluorochrome used (e.g. CD4-FITC).
- [x] The axis scales are clearly visible. Include numbers along axes only for bottom left plot of group (a 'group' is an analysis of identical markers).
- [x] All plots are contour plots with outliers or pseudocolor plots.
- [x] A numerical value for number of cells or percentage (with statistics) is provided.

Methodology

Sample preparation

For cell cycle analyses, adherent cells from tissue culture were trypsinized and fixed in 70% ethanol Cells were permeabilised in PBS containing 0.1% Triton X-100 and 1% BSA for 15 minutes. Permeabilized cells were incubated for at least 1 hour with primary antibody in PBS containing 0.1% Tween-20 and 1% BSA. The sample was washed in PBS containing 0.1% Tween 20. The cells were next incubated for at least 30 min with the corresponding secondary antibody in PBS containing 0.1% Tween-20 and 1% BSA. After washing in PBS containing 0.1% Tween 20, cells were stained for at least 30 min in PBS with 0.1% BSA, 0.1 mg/ml RNAse A and 2 µg/ml propidium iodide. After washing in PBS containing 0.1% Tween 20, the sample was resuspended in PBS with 0.1% BSA and analysed. For cell proliferation analyses, cells were stained following the manufacturer's protocol (Cell TraceTM Cell Proliferation kit, ThermoFisher Scientific, C34564). Briefly, cells were incubated in 0.5 µM Cell Trace Far red solution at 37 oC for 20 min in PBS. Four volumes of complete medium were added, and cells were further incubated for 5 min at 37 oC. Unbound dye was removed, and cells were seeded in fresh complete medium. Cells were collected by trypsinization and stained with DAPI (50 ng/ml) to distinguish live and dead cells.

Instrument	Cell cycle analyses was performed on FACSCalibur (Becton Dickinson) equipped with CellQuest Pro software (version 6.0) or CytoFLEX LX (Becton Dickinson) equipped with the CytExpert programme (version 2.3.0.84). Cell Trace Cell proliferation assay was conducted using Cytek DxP8 Flow cytometer equipped with FlowJo CE software (version 7.5.110.7).
Software	Cell cycle distribution was assessed using FlowJo software (versions 10.5.3, 10.6.2 or 10.7.2). Cell proliferation was assessed using FlowJo software (versions 10.5.3)
Cell population abundance	Cell sorting was not employed.
Gating strategy	Excessive debris were removed from the population through forward versus side scatter (FSC vs SSC) gating where necessary, and single cells were identified through FL2A vs. FL2W gating to discriminate single cells from doublets. Gating strategy is depicted in Supplementary Fig. 7.

☒ Tick this box to confirm that a figure exemplifying the gating strategy is provided in the Supplementary Information.
